# CEP55 as a prognostic indicator and a predictive marker in oral squamous cell carcinoma

**DOI:** 10.7150/ijms.107996

**Published:** 2025-04-28

**Authors:** Ying Han, Xin Hu, Haofeng Xiong, Liujun Zeng, Ying Peng, Tong Su

**Affiliations:** 1 Department of Stomatology, Center of Stomatology, Xiangya Hospital, Central South University, Changsha, Hunan, 410008, China.; 2 Research Center of Oral and Maxillofacial Tumor, Xiangya hospital of Central South University, Changsha, Hunan, 410008, China.; 3 Institute of Oral Cancer and Precancerous Lesions, Central South University, Changsha, Hunan, 410008, China.; 4 National Clinical Research Center for Geriatric Disorders (XiangYa Hospital), Changsha, Hunan, 410008, China.; 5 Center for Medical Genetics & Hunan Key Laboratory of Medical Genetics, School of Life Sciences, Central South University, Changsha, Hunan, 410008, China.

**Keywords:** CEP55, OSCC, immune infiltration, prognosis, cell cycle

## Abstract

**Objective:** To investigate the role of CEP55 in the occurrence and development of oral squamous cell carcinoma (OSCC).

**Materials and Methods:** Through the utilization of the online OSCC database and bioinformatic analysis, we examine CEP55 expression and its correlation with prognosis, pathways, and immune infiltration. CEP55 and other biomarkers were stained using immunohistochemical methods in 57 cases of OSCC and 44 cases of adjacent paired tissues, demonstrating the clear involvement of CEP55.

**Results:** The expression levels of CEP55 were significantly higher in OSCC tissues compared to normal tissues. Additionally, higher levels of CEP55 were associated with a worse prognosis. CEP55 expression levels were significantly higher in OSCC tissues compared to normal tissues. Additionally, higher levels of CEP55 were associated with a worse prognosis. GSEA results indicated a correlation between CEP55 and the cell cycle. Immunohistochemical staining revealed a significant positive correlation between CEP55 and cell cycle-related protein markers (PCNA, P16, P21, and P53). Furthermore, CEP55 was found to significantly inhibit tumor immune infiltration. As a result, CEP55 expression decreased infiltration of 9 types of immune cells (iDC, mast cells, pDC, DC, Th17 cells, TFH, Treg, T cells, and neutrophils), while increasing infiltration of only 3 types of immune cells (Tcm, T Helper cells, and Th2 cells).

**Conclusion:** The results suggest that CEP55 plays a crucial role in the progression of OSCC promoting cell cycle progression and suppressing immune infiltration.

## Introduction

Oral squamous cell carcinoma (OSCC) is the most common malignant tumor in the oral and maxillofacial region, accounting for 90% of oral cancer cases 1. It is a significant global health concern, ranking sixth among malignant tumors worldwide 2. OSCC predominantly affects middle-aged men aged 40-60 years old (those who are heavy smokers and alcoholics) 3. It commonly occurs in facial locations such as the tongue, cheeks, gums, and maxillary sinuses 4. OSCC is a highly invasive cancer that often metastasizes to regional lymph nodes in its early stages and can progress to distant metastasis in later stages, resulting in a poor prognosis 5. However, with proper treatment and management, the 5-year survival rate can be improved 6. It is worth noting that OSCC has a high recurrence rate. Despite some advancements in diagnosis and treatment, surgery remains the primary treatment for OSCC, with radiotherapy and chemotherapy as supplementary options. Hence, investigating the molecular patterns of OSCC through large-scale sequencing and identifying significant markers for early prevention and precise treatment of OSCC is crucial.

CEP55, encoded by centrosomal protein 55, plays a crucial role in recruiting endosomal sorting complex required for transport (ESCRT) during abscission, the final step of cytokinesis 7. The CEP55 protein consists of 464 amino acids and contains four structural domains: The N-terminal Coiled Coil (CC1), the C-terminal Coiled Coil (CC2), ESCRT and ALIX binding region (EABR), and C-terminal domain 8-10. CC2 domain functions as a localization intermediate by binding to kinesin family members 23 with high affinity. EABR domain recruits endocytic complex by binding to tumor susceptibility gene 101^11^. Recently, a ubiquitin-binding domain (UBD) was identified in its C-terminal region, and this UBD is functionally required for the completion of cytokinesis by an unidentified mechanism 12. Through its two UBD regions, namely the NOA/UBAN (NEMO UBD) domain and Zinc Finger (ZF) domain, the C-terminal domain is targeted to the centrosome and midbody of cells and participates in abscission 8.

Abscission, as a crucial process in cell mitosis, determines the final outcome of cell mitosis. Alterations in the mitotic process can lead to failed cell division, resulting in growth defects, proliferative diseases, and malignant tumors 13, 14. CEP55, a coiled-coil centrosomal protein initially identified by Fabbro, is regarded as a pivotal regulator of abscission during mitotic exit. Consequently, abnormalities in CEP55 can result in cytokinesis defects and an increase in the number of cells with multiple nuclei, which can ultimately impact cell survival and contribute to the development of tumors 9. Previous studies have shown that CEP55 is overexpressed in various cancer types. CEP55 expression is increased with sequential mutations in key driver genes (APC, KRAS, TP53 and SMAD4) in colorectal cancer, and promotes tumorgenesis through up-regulation of PI3K/AKT pathway. CEP55 knockout significantly inhibits tumor growth and increases CD8^+^ T cell infiltration *in vitro* and *in vivo* 15. In esophageal squamous cell carcinoma, miR-378a-5p plays a tumor suppressive role after neoadjuvant immunotherapy by down-regulating APOC1/CEP55^16^. The expression level of CEP55 protein in glioma tissues was significantly higher than that in normal control group, and the expression level in glioma tissues increased with the increase of glioma grade 17. In addition, pan-cancer studies have shown that CEP55 is associated with poor prognosis in ACC, ACC, KIRC, KIRP, LIHC, LGG, LUAD, mesothelioma, and PAAD 15. However, at present, there is a lack of studies on CEP55 in oral cancer, and the value of CEP55 as a diagnostic and prognostic indicator of oral cancer is not clear.

In this study, we used transcriptome data from the Gene Expression Omnibus (GEO) and The Cancer Genome Atlas (TCGA) databases. First, we investigated the differential expression of CEP55 in normal tissues and OSCC tissues. Subsequently, we used bioinformatics analysis to determine the role of CEP55 in OSCC clinical progression, prognosis, biological functions, and immune infiltration. Then, immunohistochemistry (IHC) was used to detect the differences in CEP55 protein expression and mRNA levels in OSCC tissues, adjacent tissues (AT), and normal tissues. Finally, IHC was used to detect biomarkers related to proliferation, apoptosis, hypoxia, and metastasis in OSCC, and to explore the relationship between CEP55 and these biomarkers.

## Material and Methods

### Sources and downloads of data

We obtained the Head and Neck Squamous Cell Carcinoma (HNSC) Expression Matrix (level 3, HTSeq - FPKM) and basic clinical information from the UCSC Xena database (https://xenabrowser.net/datapages/). Based on the onset location of the samples in the clinical information, we deleted HNSC with onset locations in "Tonsil", "Oropharynx", "Larynx ", "Lip", "Hypoharynx", "Oropharynx" and samples with incomplete clinical data. A total of 361 OSCC samples were retained, containing 329 tumor samples and 32 normal samples. In addition, we also obtained two datasets, GSE30784 and GSE37991, from NCBI GEO database to validate the expression of CEP55. GSE30784 was sequenced using Affymetrix GPL570 sequencing platform, and contained 167 OSCC samples and 45 normal samples after removing dysplasia samples, and GSE37991 was sequenced using Illumetrix GPL570 sequencing platform, which contained 167 OSCC samples and 45 normal samples. GSE37991 using Illumina's GPL6883 sequencing platform containing 40 OSCC samples and 40 normal oral tissue samples 18.

### Comparison of CEP55 expression between OSCC samples and normal samples

To compare the differences in CEP55 expression at the mRNA level between OSCC samples and normal samples, we performed independent samples t-tests for CEP55 at the transcript level in the TCGA-OSCC, GSE30784, and GSE37991 datasets using the unpaired method and paired samples T-test. To determine the prognostic value of CEP55 expression in OSCC, we used the survminer package to perform Log-rank tests on the expression levels of CEP55 and clinical prognosis data in TCGA-OSCC. We conducted Overall Survival, Disease Specific Survival, and Progress Free Interval analyses. Additionally, we used the pROC package to perform ROC analysis to evaluate the value of CEP55 in distinguishing between normal and tumor samples 19. In order to compare the expression differences of CEP55 at the protein level between OSCC samples and normal samples, we collected 61 OSCC and 47 paired paraneoplastic samples from Xiangya Hospital from 2018 to 2019 and constructed tissue microarrays of the samples, for the collection criteria of the samples and the microarray construction method, please refer to our 2021 publication on the relationship of G3BP1 with OSCC article 20. We performed IHC staining on the constructed tissue microarray to detect CEP55. After IHC staining, image scanning was conducted by Wuhan Servicebio. Quantitative analysis of IHC was performed using ImagePro Plus software (version 6.0) to evaluate the integrated optical density (IOD).

### Screening and enrichment analysis of genes related to CEP55 mRNA levels

We divided the samples in the TCGA-OSCC dataset in half based on CEP55 expression levels, namely the high CEP55 expression group and the low CEP55 expression group. We then used the DESeq2 package to screen for significantly differentially expressed genes with |logFC| > 1 and *p*-value < 0.05 as the criteria 21. Then, Gene Ontology (GO) and Kyoto Encyclopedia of Genes and Genomes (KEGG) pathway enrichment analyses were performed. GO analysis includes three parts: Biological Process (BP), Molecular Function (MF), and Cellular Component (CC). The org.Hs.eg.db package [version 3.10.0] is used for ID conversion, and the clusterProfiler package [version 3.14.3] is used for enrichment analysis 22. Gene set enrichment analysis (GSEA) was performed to analyze the functional enrichment of CEP55 on a global scale, and the condition of significant enrichment was defined as False discovery rate (FDR) < 0.25 and p.adjust < 0.05 23.

### Analysis of CEP55 in relation to immune infiltration

To evaluate the relationship between CEP55 immune infiltration in OSCC, we used the ssGSEA algorithm. Subsequently, the enrichment scores of various immune cell subtypes within OSCC tissues were calculated to comprehensively understand the immune - infiltrating microenvironment associated with CEP55 expression. After obtaining these scores, a correlation analysis was conducted between the CEP55 expression levels and the enrichment scores of different immune cell subtypes, including activated dendritic cells (aDC) 24, 25, and compared the StromalScore, ImmuneScore and ESTIMATEScore between CEP55 high and low expression groups of TCGA-OSCC by using the ESTIMATE package [version 1.0.13]^26^. Additionally, by using immunofluorescence (IF) staining to detect the expression of immune cell markers CD3, CD4, and CD8 in tissues, we aim to evaluate the correlation between CEP55 and these immune cell markers at the protein level.

### Analysis of the relationship between CEP55 and proliferation markers

To clarify the role of CEP55 in cell proliferation, we investigated its correlation with immune cell markers at the protein level. This was achieved through generalized immunohistochemical (IHC) staining, utilizing proliferation markers such as P16, P21, P53, PCNA, TGFB1, MAPK, p-AKT, and β-catenin.

### Ethical declaration

The clinical sample collection in this study was conducted in accordance with the Declaration of Helsinki (2002 version) and additional requirements. All collected samples were obtained with written informed consent from the patients. Experiments on the samples were also conducted with the understanding and written consent of each donor. This study was approved by the Ethics Committee of the School of Life Sciences, Central South University (NO.2020-1-42). Additionally, the public sequencing data used in this study were obtained from the NCBI GEO database and the TCGA database (https://tcga-data.nci.nih.gov/tcga/tcga). The analysis and processing of the data complied with the usage norms of each database, ensuring no ethical or safety issues, and the data used are freely accessible to anyone.

### Statistical analysis

This study used R software (version 4.0.2) and related software packages for statistical analysis and visualization of results. Comparisons between the two groups were made using independent samples t-test or paired samples t-test. Pearson correlation analysis was used to analyze the correlation between the two variables. Log-rank test was used for Kaplan-Meier's analysis. All results were defined as significant with a p-value less than 0.05.

## Results

### CEP55 was significantly overexpressed in OSCC samples

**Figure 1:**
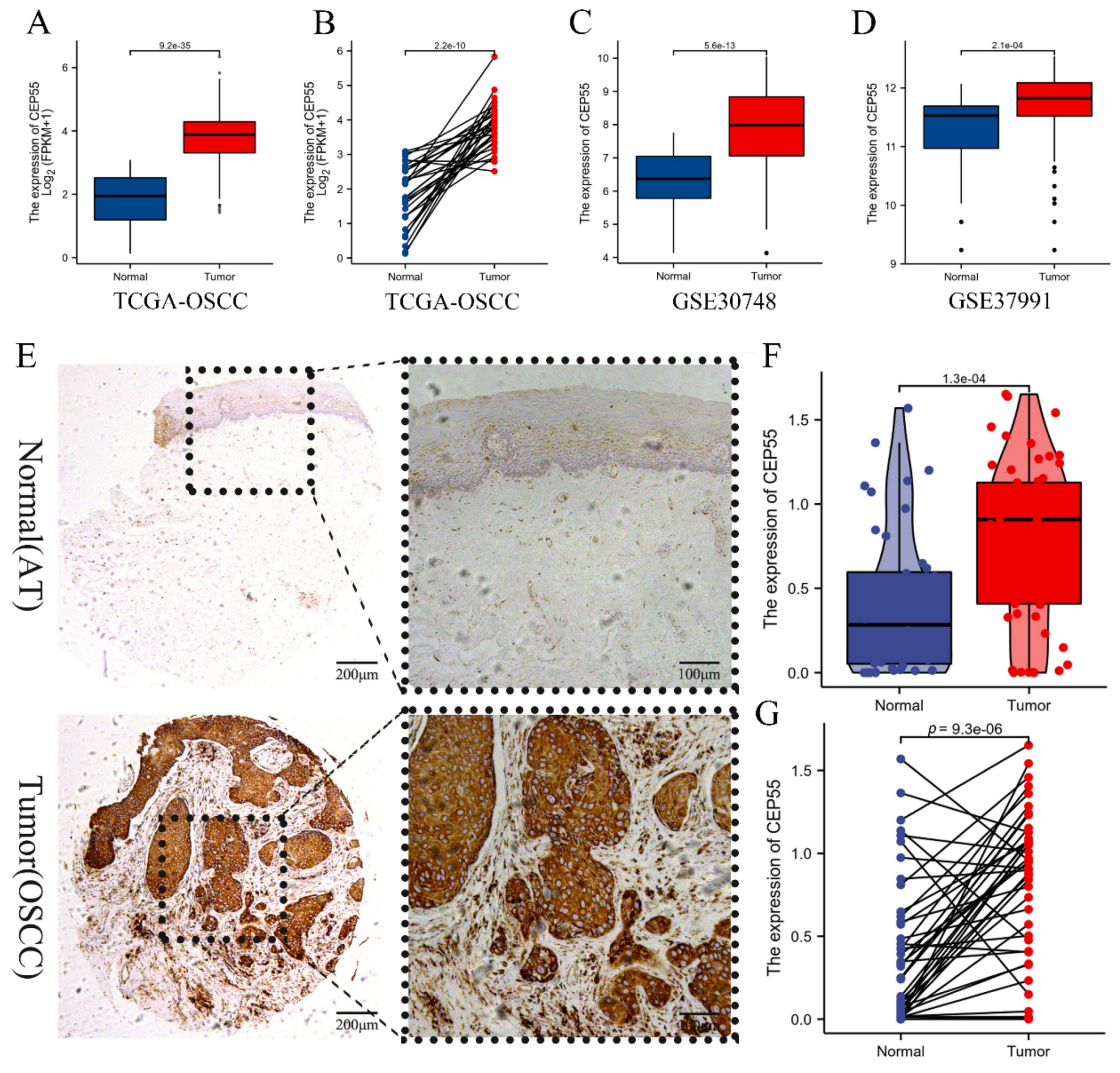
** Comparison of CEP55 between OSCC and normal samples.** (A-B) Comparison of CEP55 mRNA levels between normal and OSCC tissue samples as a whole (A) and after sample pairing (B) in TCGA database, unpaired t-test vs. paired t-test, respectively. (C-D) Comparison of CEP55 mRNA levels between normal and OSCC tissue samples as a whole in the GEO and TCGA databases (C. GSE30748; D. GSE37991), unpaired t-test. (E) Paired IHC images of CEP55 in OSCC and paracancerous tissues. (F-G) Unpaired t-test versus paired t-test of CEP55 protein levels between OSCC and paracancerous tissues.

We first compared TCGA-OSCC mRNA expression levels of CEP55 between tumor and normal samples, and the results showed that the mRNA levels of CEP55 were significantly upregulated in tumor samples (Figure 1A), and the results of paired t-tests also showed that the mRNA expression levels of CEP55 were high in OSCC samples (Figure 1B). To verify TCGA that the mRNA expression level of CEP55 was significantly elevated in OSCC samples, we then analyzed the mRNA expression of CEP55 in the GSE30784 and GSE37991 datasets, and the results were consistent with those of the TCGA-OSCC analysis (Figure 1C-D). To further investigate the protein expression of CEP55 was also up-regulated in OSCC, we immunohistochemically quantified CEP55 protein in clinical samples, which also showed that the expression of CEP55 was significantly higher in OSCC than in paracancerous tissues (Figure 1E-G), which was consistent with our results based on the analyses of multiple public datasets. All the findings suggest that CEP55 expression is over-activated in OSCC.

### The high expression of CEP55 is associated with poor prognosis

To ascertain whether CEP55 can be utilized as a prognostic marker for OSCC, we conducted a survival analysis of TCGA-OSCC, which demonstrated that elevated CEP55 expression in TCGA-OSCC was associated with poor overall survival (OS) prognosis (HR=1.52 (1.10-2.20), Log-rank *p*=0.011) and also with poor disease-specific survival (DSS). The results indicated that CEP55 expression was associated with reduced disease-specific survival (DSS) (HR=1.50 (1.03-2.34), Log-rank *p*=0.021), and also with poor progression-free interval survival (PFI) (HR=1.31 (0.93-1.84), Log-rank p=0.047). Furthermore, the difference in the degree of elevation of CEP55 in OSCC was investigated by applying the ROC curve to determine the differential diagnostic value of CEP55 in OSCC. The ROC curve analysis demonstrated that CEP55 exhibited an AUC value of The ROC curve analysis demonstrated that CEP55 exhibited an AUC value of 0.967 (95% CI: 0.948-0.986). At a cutoff value of 3.095, CEP55 demonstrated a sensitivity and specificity of 1 and 0.827, respectively. This suggests that CEP55 has a high diagnostic value in the differential diagnosis of OSCC.

### CEP55 promotes cell cycle progression

In order to explore what role CEP55 plays in OSCC, we grouped the samples in the TCGA-OSCC dataset in half according to the expression of CEP55 and screened the related genes for functional enrichment analysis. Among them, GO analysis revealed that biological process (BP) was mainly enriched in keratinization, keratinocyte differentiation, epidermal cell differentiation, skin development and epidermis development. And molecular function (MF) was mainly enriched in structural constituent of epidermis, structural constituent of muscle, lipase activity, calcium-dependent phospholipase A2 activity and serine-type endopeptidase inhibitor activity. Meanwhile, the cellular component (CC) is mainly enriched in cornified envelope, sarcomere, contractile fiber part, contractile fiber and myofibril. In addition to this, KEGG is mainly enriched in numerous metabolic processes, including Arachidonic acid metabolism, Linoleic acid metabolism, alpha-Linolenic acid metabolism, Ether lipid metabolism and Sphingolipid metabolism. Concurrently, to globally examine the function of CEP55, we conducted a GSEA analysis, which revealed that elevated CEP55 expression markedly activated KEGG CELL CYCLE, KEGG ONE CARBON POOL BY FOLATE, KEGG MISMATCH REPAIR, and KEGG HOMOLOGOUS RECOMBINATION. Furthermore, KEGG DNA REPLICATION was found to significantly suppress KEGG NEUROACTIVE LIGAND RECEPTOR INTERACTION, KEGG FOCAL ADHESION, KEGG CALCIUM SIGNALING PATHWAY, KEGG ALZHEIMERS DISEASE, and KEGG XIDATIVE HOSPHORYLATION.

### CEP55 impedes the infiltration of immune cells into tumors

**Figure 4:**
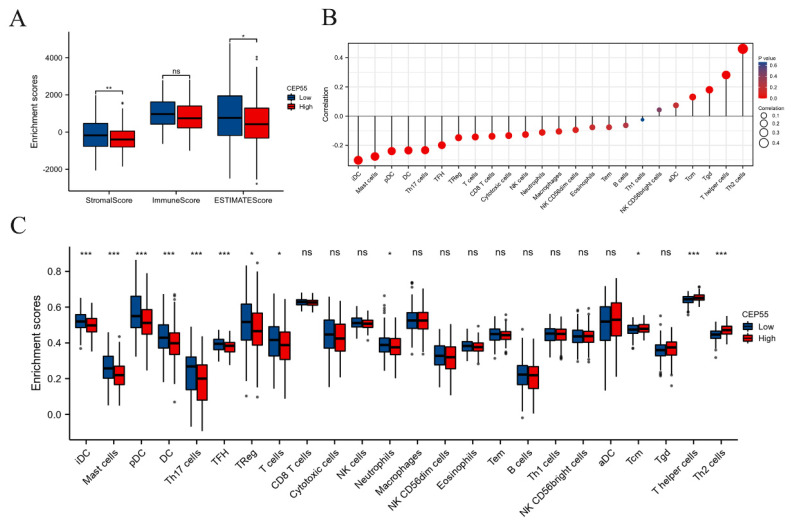
** CEP55 expression and tumor immune infiltration.** To this end, we have analyzed the stromalScore, immuneScore, and ESTIMATEScore of CEP55 expression in high and low expression groups. Furthermore, we have investigated the relationship between CEP55 and the infiltration level of various immune cells. (A) StromalScore, ImmuneScore, and ESTIMATEScore of the high and low expression groups of CEP55, * *p* < 0.05, ** *p* < 0.01, ns= no significance. (B-C) Correlation between CEP55 expression and the infiltration level of various immune cells, * *p* < 0.05, *** *p* < 0.001, ns= no significance.

The tumor microenvironment (TME) is a critical factor in the development and progression of OSCC. To investigate the function of CEP55 in immune infiltration, we conducted a comparative analysis of the StromalScore, ImmuneScore, and ESTIMATEScore between the high and low CEP55 expression groups in TCGA-OSCC. The results demonstrated that StromalScore, ImmuneScore, and ESTIMATEScore exhibited diminished scores in the CEP55 high-expression cohort, indicating that CEP55 was impeding immune infiltration (Figure 4A). To validate this finding, we conducted a systematic investigation of the correlation between CEP55 and 24 immune cells using ssGSEA. The results demonstrated a significant positive correlation between CEP55 expression in OSCC and the abundance of Th2 and Th17 cells, which inhibit immune infiltration, and a negative correlation between CEP55 expression and the abundance of iDC, mast cell, pDC, and DCs that activate immune infiltration. The abundance of other immune cells exhibited no strong correlation (Figure 4B-C). These findings align with those of our tissue microarray.

### CEP55 is associated with cell proliferation

**Figure 5:**
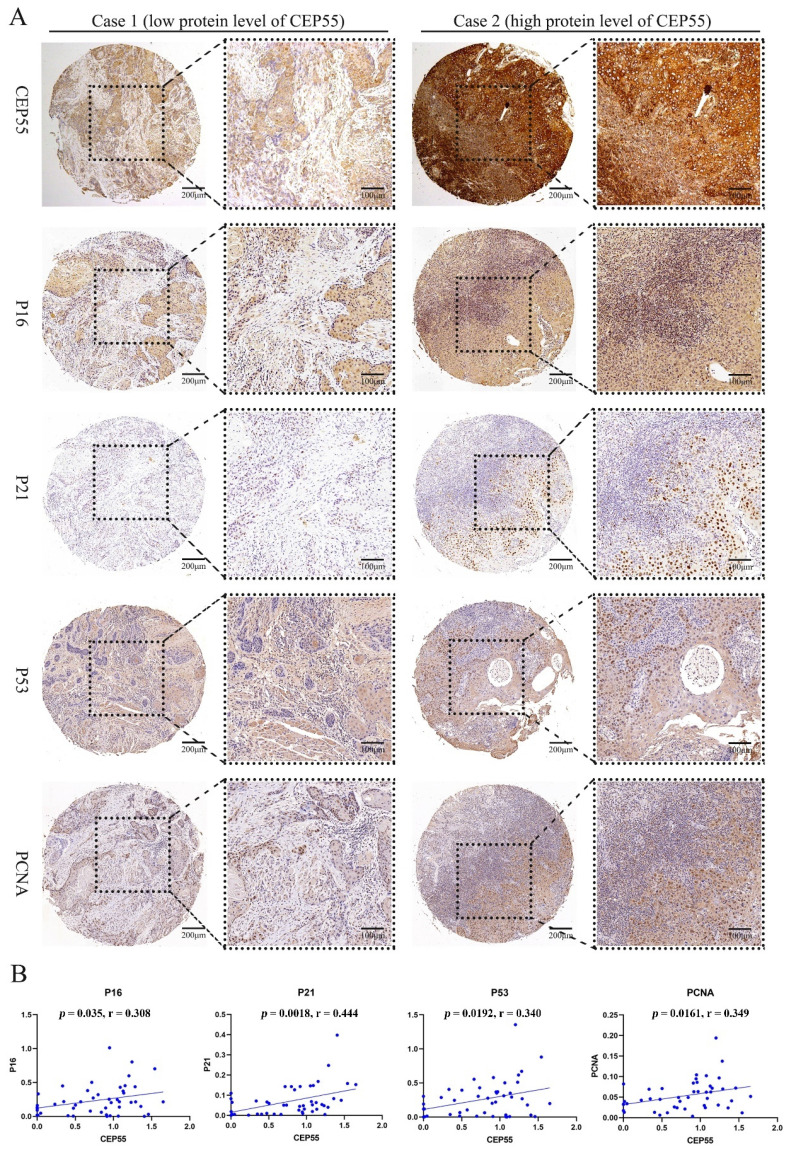
** CEP55 and OSCC Cell Proliferation Markers P16, P21, P53, and PCNA.** (A) IHC images of OSCC tissues in the high and low CEP55 expression groups. The IHC staining markers include CEP55 and proliferation markers P16, P21, P53, and PCNA. (B) Unpaired T-test of the protein levels of proliferation markers in OSCC tissues between the high and low CEP55 expression groups.

**Figure 6:**
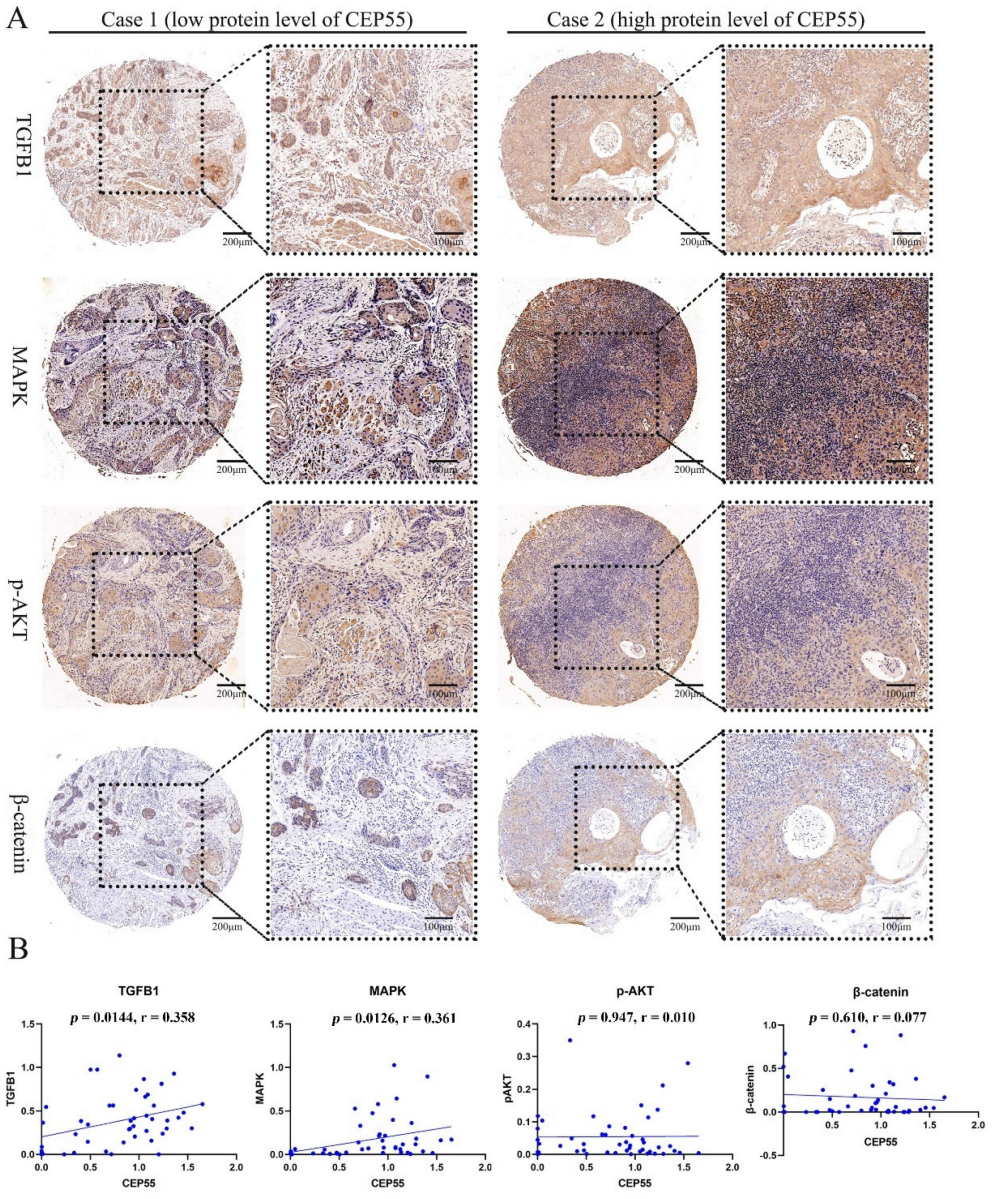
** CEP55 and OSCC Cell Proliferation Markers TGFB1, MAPK, p-AKT, and β-catenin.** (A) IHC images of OSCC tissues in the high and low CEP55 expression groups. The IHC staining markers include CEP55 and TGFB1, MAPK, p-AKT, and β-catenin. (B) Unpaired T-test of the protein levels of TGFB1, MAPK, p-AKT, and β-catenin in OSCC tissues between the high and low CEP55 expression groups.

To ascertain the function of CEP55 and determine its relationship to the cell cycle, we conducted a comparative analysis of the differences between the high and low expression groups of CEP55 protein in tissue microarrays. The results demonstrated that the IHC staining of P16, P21, P53, and PCNA was more profound in the CEP55 protein expression group (Figure 5A). The results demonstrated a significant correlation between CEP55 and P16, P21, P53, and PCNA. Among these, the correlation between CEP55 and P16 was the most significant (cor = 0.308, *p* = 0.035), followed by that between CEP55 and P21 (cor = 0.444, *p* = 0.0018), P53 (cor = 0.340, *p* = 0.0192), and PCNA (cor = 0.349, *p* = 0.0161). (Figure 5B). Furthermore, we conducted a comparative analysis of the differences in TGFB1, MAPK, p-AKT, and β-catenin expression between the high and low CEP55 protein expression groups in tissue microarrays. Additionally, we compared the differences in TGFB1, MAPK, p-AKT, and β-catenin between the high and low CEP55 protein expression groups in tissue microarrays. Although TGFB1, MAPK, p-AKT, and β-catenin are more highly expressed in the high CEP55 expression group (Figure 6A), CEP55 is significantly positively correlated only with TGFB1 (cor = 0.358, *p* = 0.0144) and MAPK (cor = 0.361, *p* = 0.0126), and not significantly correlated with p-AKT (cor = 0.010, *p* = 0.947) and β-catenin (cor = 0.077, *p* = 0.610) (Figure 6B). These results suggest that CEP55 may promote cell proliferation in OSCC through P16, P21, P53, PCNA, TGFB1, and MAPK.

### CEP55 is related to tumor immune status

**Figure 7:**
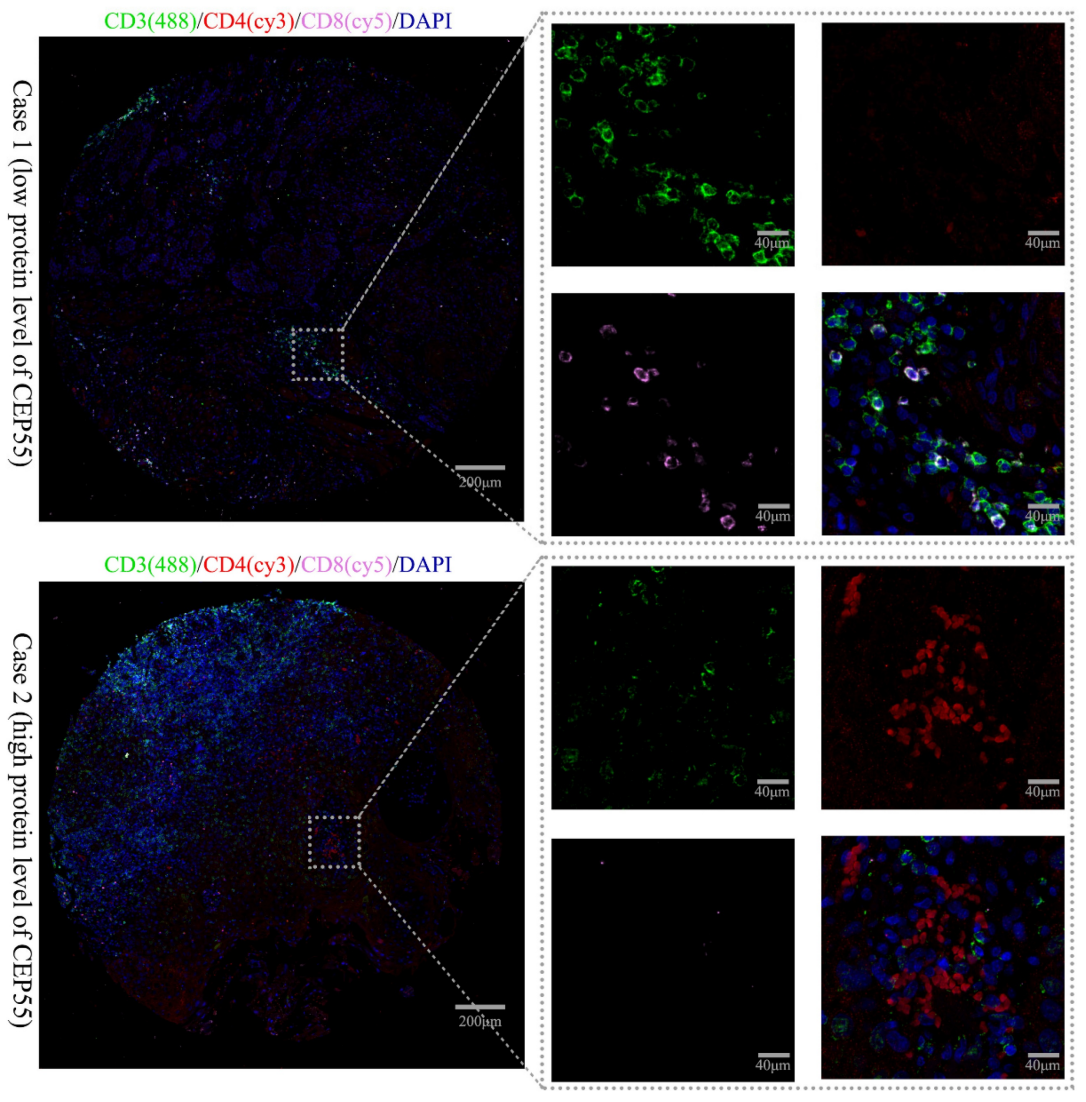
** IP images of OSCC tissues in the high and low CEP55 expression groups.** The IP staining markers include immune phenotype markers CD3 (green), CD4 (red), CD8 (pink), and DAPI (blue). The scale bar is indicated in the figure.

This study also explored the relationship between CEP55 expression and T cell lymphocyte subpopulations using triple immunofluorescence staining. The immune phenotype of T lymphocytes was evaluated by IHC. Patients with low CEP55 expression predominantly had CD8^+^ T cell infiltration, while patients with high CEP55 expression predominantly had CD4^+^ T cell infiltration (Figure 7). This is consistent with previous bioinformatics analysis results, which showed that CEP55 expression is associated with the inhibition of immune cell infiltration.

## Discussion

At present, radical surgical resection represents the primary treatment modality for OSCC. While surgical resection can remove lesion tissue and inhibit disease progression, it often results in significant defects in maxillofacial tissue. Furthermore, OSCC exhibits strong local invasiveness, increasing the likelihood of lymphatic metastasis and, consequently, high metastasis and recurrence rates. Consequently, the prognosis of surgical treatment is not favorable. Therefore, identifying more effective biomarkers for early screening of OSCC and as targets for precise treatment at the disease onset will be a crucial area of focus.

CEP55, encoded by the centrosomal protein 55 gene, plays a pivotal role in recruiting the endosomal sorting complex required for transport (ESCRT) during abscission, the final stage of cell mitosis 7. The CEP55 protein is comprised of 464 amino acids and contains four domains: the N-terminal coiled-coil, the C-terminal coiled-coil, the EABR region, and the C-terminal domain. The C-terminal coiled-coil domain interacts with kinesin family member 23 to facilitate the localization of the midbody. And the C-terminal domain, through its NOA/UBAN domain and zinc finger structure, participates in abscission and midbody localization 8-10. Abscission is a critical process in cell mitosis, and its proper progression is essential for the successful completion of cell mitosis. Therefore, when the key factor controlling this process, CEP55, is abnormal, it will lead to defective cytokinesis and an increase in the number of multinucleated cells, which will ultimately affect cell survival and promote tumorigenesis 13, 14.

In some cancers, such as non-small cell lung cancer 27, pancreatic cancer 28, renal cell carcinoma 29, and T cell lymphoma 30, CEP55 is often highly expressed and promotes tumor development, thereby becoming an effective diagnostic marker. Nevertheless, the function of CEP55 in OSCC remains largely uncharacterized. In this study, we investigated the expression of CEP55 in OSCC for the first time and its impact on OSCC prognosis. Our findings indicate that CEP55 expression is elevated in OSCC at both the transcriptional and protein levels. Furthermore, our findings suggest that high expression of CEP55 may be associated with a poor prognosis in OSCC. Our results also showed that CEP55 has high sensitivity and specificity in OSCC. High sensitivity means that a large proportion of OSCC patients can be detected by measuring CEP55 expression, reducing the number of false-negative cases. High specificity ensures that the test does not misclassify non-OSCC patients as having the disease, minimizing false-positive results. These findings are consistent with those observed in other cancers, indicating that CEP55 may serve as a promising prognostic and diagnostic marker.

Further in-depth analysis via GSEA revealed that the role played by CEP55 in OSCC appears to be intimately connected with the cell cycle. Studies in other cancers have also shown that CEP55 primarily promotes tumorigenesis by affecting cell proliferation, apoptosis, migration, and invasion. Specifically, high expression of CEP55 increases cell proliferation and colony formation abilities, significantly reduces apoptosis, and enhances cell migration and invasion capabilities 31, 32. Consequently, immunohistochemistry was employed to investigate the correlation between CEP55 and several main cell cycle markers. Notably, CEP55 exhibits a positive correlation with the proliferation marker PCNA and three additional proteins associated with cell cycle arrest, namely P53, P16, and P21. In some studies, the simultaneous positivity of P53, P16, and P21 typically indicates that cells have entered a state of cell cycle arrest, and therefore, these markers are often used as indicators of cellular senescence 33. In this study, we found that the positive distribution of P53, P16, and P21 was consistent with the high expression of CEP55 in OSCC tissues through serial paraffin sections and IHC staining.

It is noteworthy that, upon PCNA staining, these cells did not enter a senescent state; instead, they exhibited heightened proliferative activity. These findings may appear contradictory, but this is not the case in OSCC. It has been demonstrated that cells exhibiting positive P53 expression demonstrate heightened proliferative activity in OSCC, which is indicative of a poor prognosis 34, 35. In a study conducted by Geo Francis and colleagues, it was observed that despite the accumulation of P53 in OSCC cells, these proteins tend to remain inactive 36. Judit A Nemes *et al.* found that positive expression of P21 is associated with tumor size and lymph node involvement, is positively correlated with the proliferation marker Ki67, and may indicate poor prognosis in OSCC 37. The impact of P16 on prognosis is controversial. Reports by Alexander *et al.* 38 and Katrine *et al.* 39 suggest that P16 does not affect the prognosis of OSCC patients, while studies by Niklas *et al.* 40 and Swati *et al.* 41 indicate that patients with low P16 expression have a poor prognosis. A possible explanation is that CEP55 may promote the proliferation of OSCC cells by inhibiting the functions of P53/P21/P16, while the accumulation of P53/P21/P16 in OSCC cells may be related to a negative feedback mechanism in intracellular signal regulation.

Additionally, we investigated the relationship between CEP55 and several key cancer-related pathways. Our findings revealed that CEP55 expression is positively correlated with the expression of TGFB1 and MAPK. TGFB1 can directly participate in the invasion and metastasis of OSCC 42. Meanwhile, the MAPK signaling pathway is closely related to the proliferation and apoptosis of OSCC 43. This finding is consistent with the overexpression of CEP55 in OSCC and the poor prognosis, indicating that CEP55 can directly promote the occurrence and development of OSCC through the TGFB1 pathway or MAPK pathway. Additionally, TGFB1 plays a significant role in immune regulation. TGFB1 induces the infiltration of Tregs and cancer-associated fibroblasts, thereby inhibiting the cytotoxic T cell and NK cell killing, creating an immunosuppressive tumor microenvironment 44. It can thus be postulated that the function of CEP55 in OSCC may be related to the immunomodulatory effect of TGFB1.

The infiltration of immune cells in tumors is closely related to the efficacy of clinical immunotherapy. The immune cell infiltration pattern in oral squamous cell carcinoma (OSCC) has significant clinical implications. Higher infiltration of cytotoxic T cells, such as CD8^+^ T cells, is generally associated with a better prognosis in OSCC. These cells have the ability to recognize and kill tumor cells directly, which may lead to tumor regression and longer patient survival. In contrast, increased infiltration of regulatory T cells (Tregs) or myeloid - derived suppressor cells (MDSCs) is often associated with a poorer prognosis. Tregs can suppress the anti-tumor immune response by inhibiting the activation and function of effector T cells, while MDSCs can promote an immunosuppressive microenvironment and support tumor growth and metastasis. However, the relationship between CEP55 and tumor immune infiltration in OSCC remains unclear. By evaluating the differences in 24 types of immune cells between high and low CEP55 expression groups, it was found that CEP55 significantly inhibits tumor immune infiltration. Specifically, the infiltration levels of nine types (37.5%) of immune cells, including iDCs, Mast cells, pDCs, DCs, Th17 cells, Tfh cells, Tregs, T cells, and Neutrophils, decreased with increased CEP55 expression. Conversely, the infiltration levels of only three types (12.5%) of immune cells increased, including Tcms (Central Memory T cells), T helper cells, and Th2 cells. These results suggest that CEP55 may play a role in regulating the tumor microenvironment. High CEP55 expression and increased infiltration of Tregs lead to an immunosuppressive microenvironment, reducing the efficacy of immunotherapy.

IHC analysis also revealed that in patients with low CEP55 expression, the lymphocyte subpopulation infiltration in the tumor microenvironment was predominantly CD8^+^ cells, whereas in patients with high CEP55 expression, the infiltration was predominantly CD4^+^ cells. Geetashree *et al.* found that patients with high levels of CD8^+^ T cell infiltration had better prognoses 45. Similarly, Joabe *et al.* reported that in OSCC, patients with a higher ratio of CD8 to CD4^+^ cells had better prognoses 46. These findings are consistent with the conclusions of this study, further demonstrating that high CEP55 expression is associated with an immunosuppressive state of tumor microenvironment. However, further studies on the relationship between CEP55 expression and the tumor microenvironment are still needed to determine whether CEP55 can be used as a biomarker to predict OSCC immunotherapy response.

In this study, we employed a combination of bioinformatics analysis and tissue microarray immunohistochemistry to investigate the expression and function of CEP55 in OSCC. Our findings indicate that CEP55 is associated with OSCC proliferation (in terms of the cell cycle) and immune infiltration, thereby underscoring the potential value of CEP55 as a diagnostic and prognostic marker for OSCC. Nevertheless, it should be acknowledged that the study is not without limitations. Firstly, it is inevitable that there will be a degree of selection bias. Secondly, the OSCC tissue samples used for immunohistochemistry were all derived from Asian populations, which may introduce potential biases due to ethnic differences that were not accounted for. Ultimately, further large-scale cohort studies are required to ascertain the specificity and accuracy of CEP55 as a clinical biomarker.

## Figures and Tables

**Figure 2:**
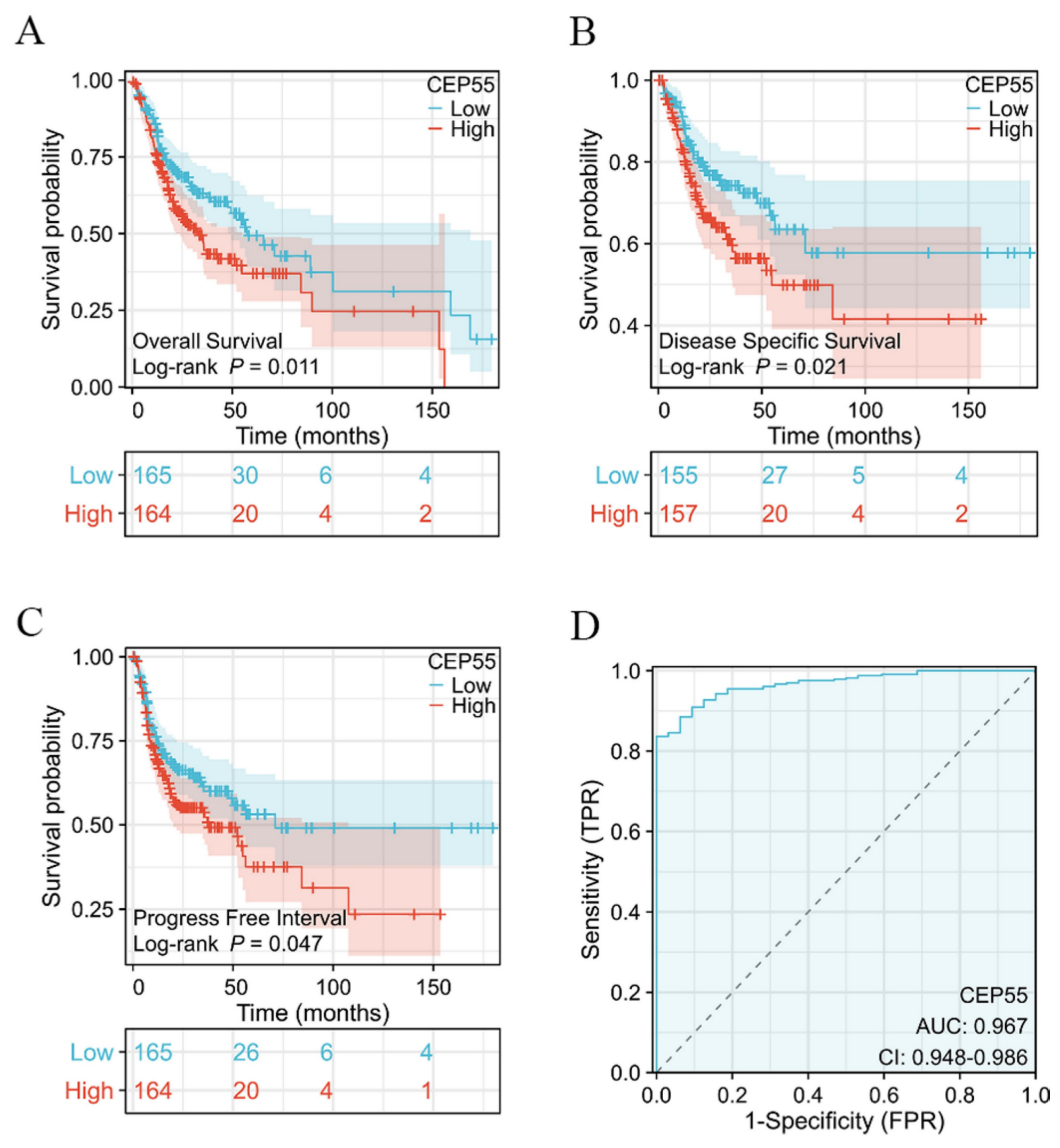
** Survival analysis based on CEP55 mRNA levels.** (A-C) Overall survival (A), disease-specific survival (B), and progression-free interval survival (C) in the CEP55 high-expression group versus the low-expression group, Log-rank test. (D) ROC curves for the variability of the degree of CEP55 elevation in OSCC.

**Figure 3:**
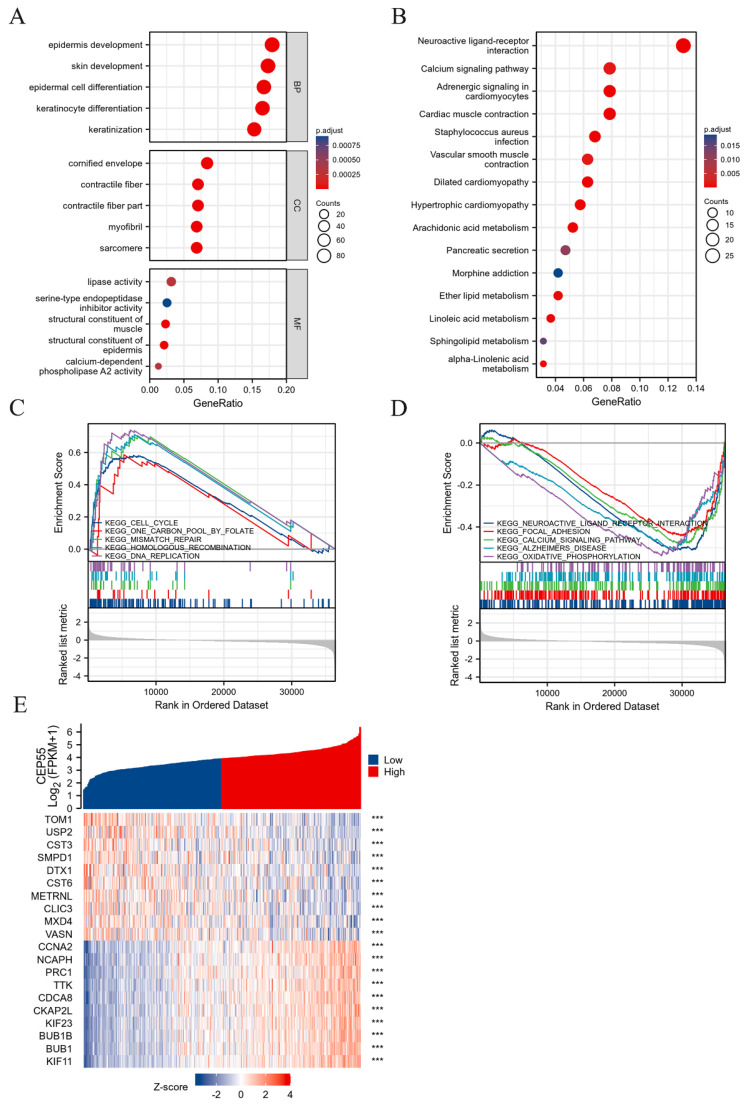
** Screening and enrichment analysis of differentially expressed genes (DEGs) between high and low expression groups divided by CEP55 mRNA levels.** (A-B) Gene ontology (GO) and Kyoto Encyclopedia of Genes and Genomes (KEGG) Analyses of Differentially Expressed Genes in OSCC CEP55 High and Low Expression Groups. (C-D) Gene Set Enrichment Analysis (GSEA) Showing Activation and Inhibition Pathways of CEP55 High Expression in OSCC. (E) Heatmap of CEP55 Up-regulated Differentially Expressed Genes (DEGs) and Down-regulated Differentially Expressed Genes in OSCC, *** *p* < 0.001.
